# Supplementary material for: Integrative genome-scale analyses reveal post-transcriptional signatures of early human small intestinal development in a directed differentiation organoid model
Source: BMC Genomics. 2023 Oct 26;24:641. doi: 10.1186/s12864-023-09743-1 (PMC10601309; doi:10.1186/s12864-023-09743-1)
Supplement: Supplementary file 6 — Supplementary Material 6 [file 12864_2023_9743_MOESM6_ESM.pdf]

Supplementary Figure 1.

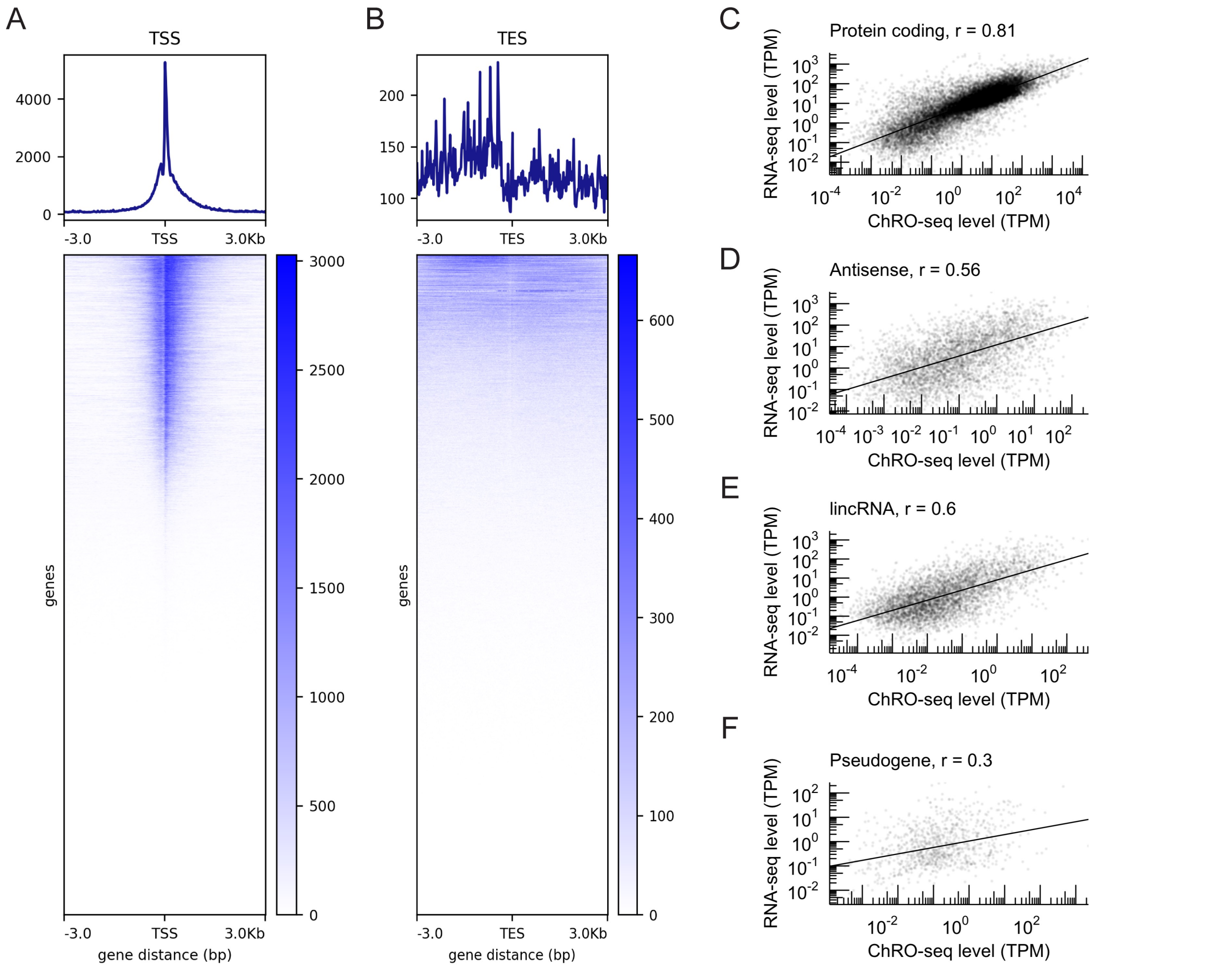

Supplementary Figure 1. Correlation analysis between ChRO-seq and RNA-seq in the hPSC-HIO model. (A) ChRO-seq intensity around transcription start sites (TSSs). (B) ChRO-seq intensity around transcription termination sites (TESs). ChRO-seq signal per stage was normalized by total bigwig signal of 10^7 before merging all four stages for this analysis. (C-F) Spearman correlation between ChRO-seq (TPM) and RNA-seq (TPM) across different gene classes, including protein coding (C), antisense (D), lncRNA (E) and pseudogene (F). In (C-F), ChRO-seq reads within 150 bp window of TSS downstream was excluded for TPM calculation and genes of which gene body is shorter than 1 kb were excluded. ChRO-seq study: hESC, n = 3; DE, n = 4; Duo spheroid (Duo), n = 3; Ile spheroid (Ile), n = 3. RNA-seq study: hESC, n = 2; DE, n = 3; Duo, n = 6; Ile, n = 4.

Supplementary Figure 2.

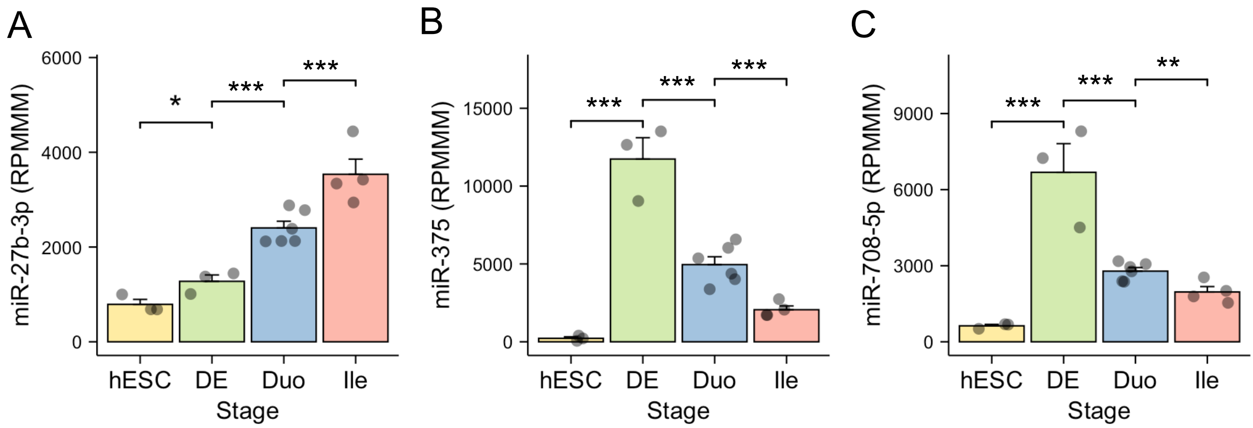

Supplementary Figure 2. MiRNAs that are significantly altered in all the stage transitions during directed differentiation process. (A) Expression level of miR-27b-3p, (B) miR-375 and (C) miR-708-5p across all 4 cell stages of the directed differentiation mode. * Padj < 0.05, ** Padj < 0.01, *** Padj < 0.001 by DESeq2 Wald test in smRNA-seq. RPMMM, reads per million mapped to miRNAs. smRNA-seq: human embryonic stem cell (hESC), n = 3; definitive endoderm (DE), n = 3; Duo spheroid (Duo), n = 6; Ile spheroid (Ile), n = 4.

Supplementary Figure 3.

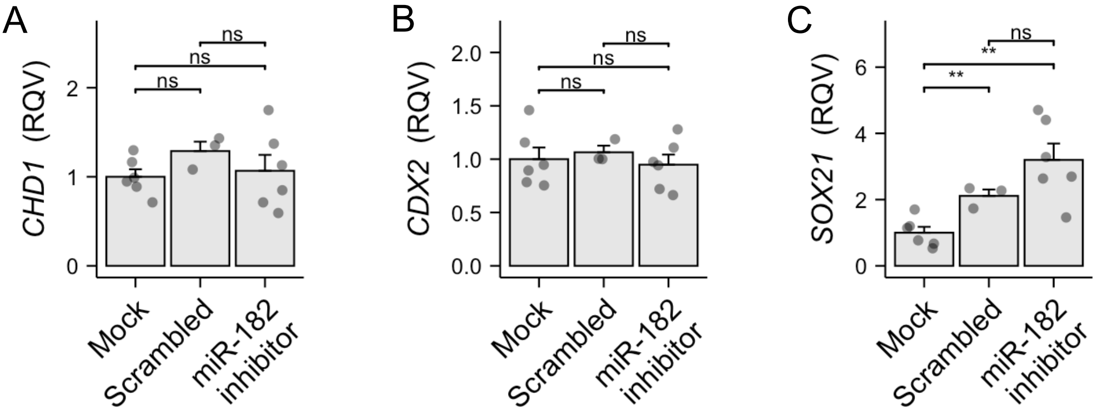

Supplementary Figure 3. Gene expression of *CHD1, CDX2* and *SOX21* in iPSC directed differentiated cells receiving treatment of mock, scrambled control or miR-182 inhibitors. (A) qPCR of epithelial marker *CHD1,* (B) hindgut marker *CDX2* and (C) SOX21 in iPSC directed differentiated cells receiving 24-hr treatment of mock, scrambled control or miR-182 inhibitors. N = 3-6 per condition across 2 independent experiments. RQV, relative quantitative value.

Supplementary Figure 4.

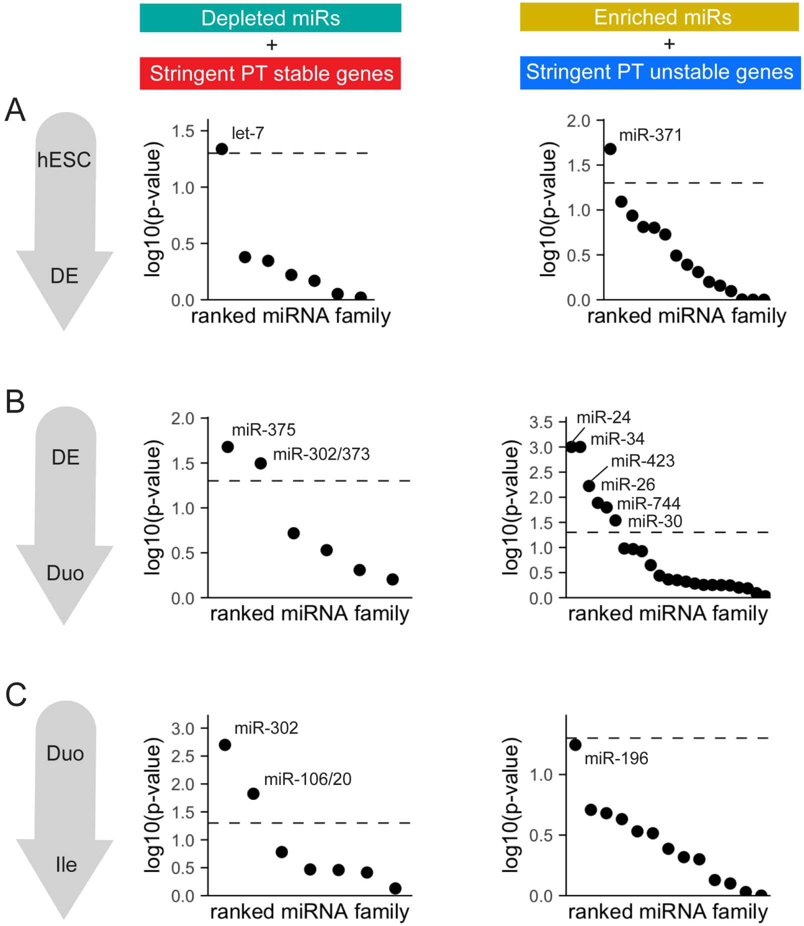

Supplementary Figure 4. miRhub analyses identified candidate miRNA regulators in the hPSC-HIO model. (A-C) MiRhub analyses assessing the impacts of depleted miRNAs on the stable genes (left panel) and the impacts of enriched miRNAs on the unstable genes (left panel) during the event of DE formation (A), SI lineage formation (B) and ileal specification (C). Dash line denotes P-value = 0.05 in miRhub analyses.

Supplementary Figure 5.

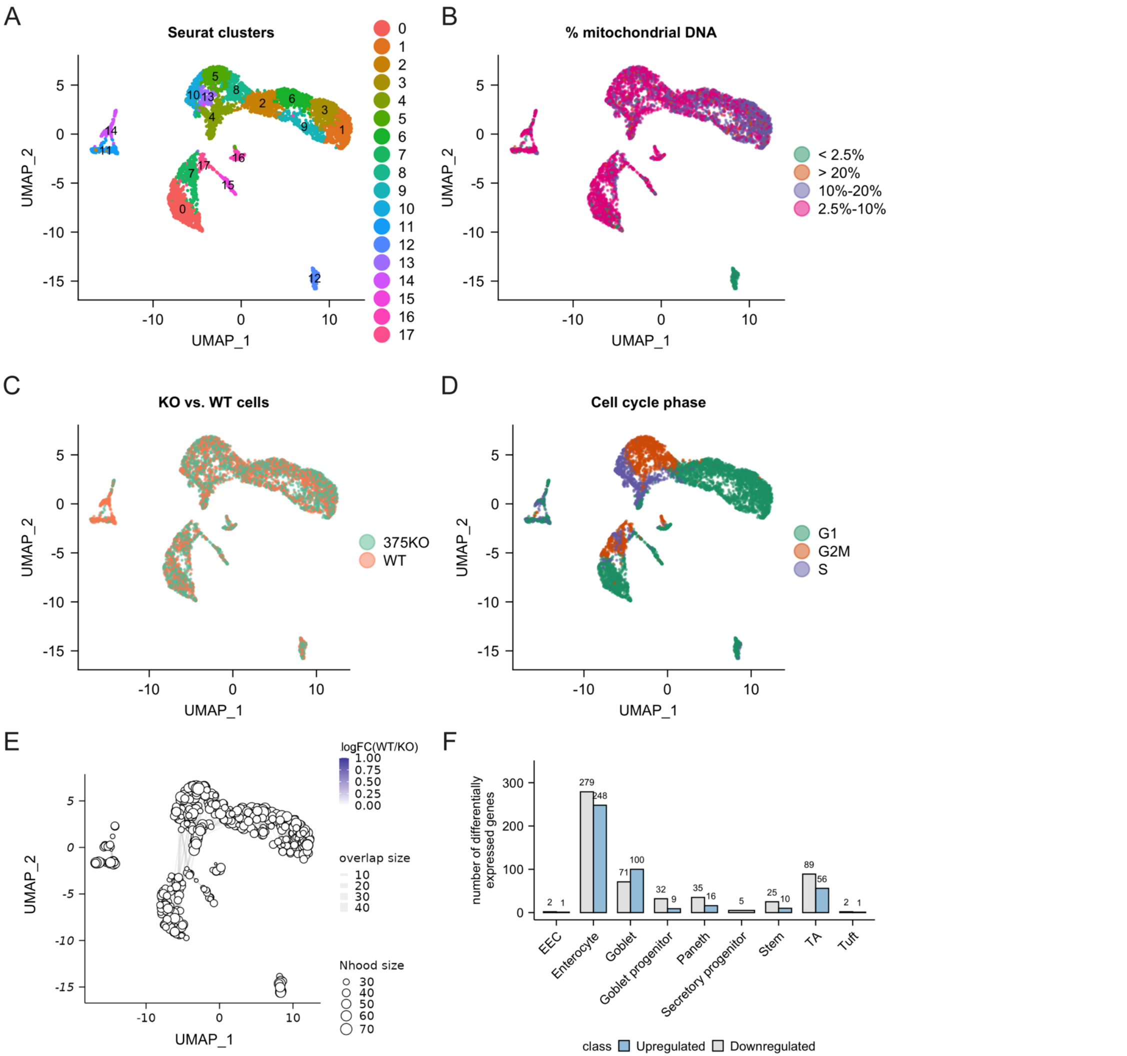

Supplementary Figure 5. scRNA-seq analyses of the intestinal epithelial cells from adult WT and 375KO mice. (A) UMAP of initial clustering analysis with intestinal epithelial cells of adult WT and whole body miR-375 knockout (375KO) mice. (B) UMAP overlain with % mitochondrial genes in the dataset. (C) UMAP overlain with genotype information (WT vs. 375KO) in the dataset. (D) UMAP overlain with cell cycle phase in the dataset. (E) Differential abundance test (using MiloR) showing similar cell type composition between WT and 375KO mice in the dataset. (F) Numbers of differential expressed genes between WT and 375KO for each cell cluster in the dataset.

Supplementary Figure 6.

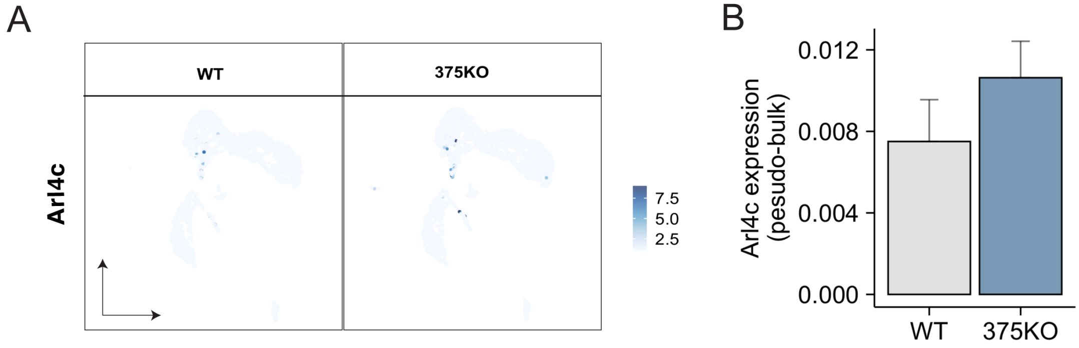

Supplementary Figure 6. Low expression of *Arl4c* in the intestinal epithelial cells from adult WT and 375KO mice. (A) UMAP overlain of *Arl4c* expression in the intestinal epithelial cells of adult WT and whole body miR-375 knockout (375KO) mice. (B) Pseudo-bulk analysis showing a modest elevation of Arl4c in 375KO (n = 2) compared to WT (n = 2) mice (1.42 fold increase, P = 0.37 by two-tailed t-test).

Supplementary Figure 7.

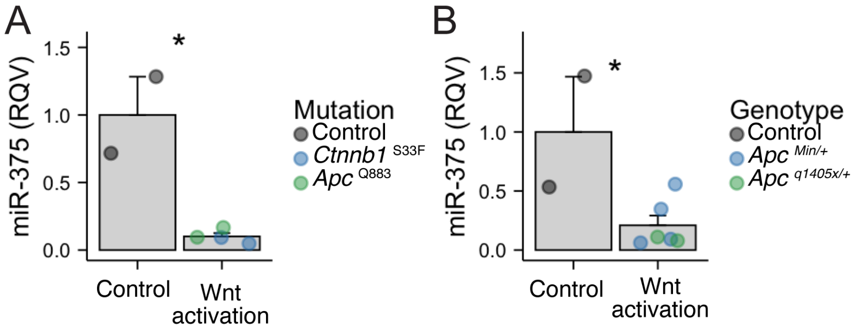

Supplementary Figure 7. Activation of Wnt signaling leads to a reduction of miR-375 expression in mouse enteroids. (A) qPCR showing *ex vivo* downregulation of miR-375 in mouse enteroids with mutation-induced Wnt activation (n = 4 including 2 wells with Apc^{Q883} mutation and 2 wells with $Ctnnb1^{S33F}$ mutation) compared to wild type control (n = 2 wells). (B) qPCR showing in vivo downregulation of miR-375 in polyps of Apc mutant mice (n = 6 tissue samples from 4 $Apc^{Min/+}$ mice and 2 $Apc^{q1405x/+}$ mice) compared to the villi scrappings of control group (n = 2 mice). APC, Adenomatous polyposis coli. *P < 0.05 by two-tailed t-test.
